# Process quality of type 2 diabetes mellitus care and association with patient perceived attributes of family doctor service in urban general practices, Beijing, China

**DOI:** 10.1186/s12875-022-01838-0

**Published:** 2022-09-07

**Authors:** Feiyue Wang, Yun Wei, Meirong Wang, Zhaolu Pan, Guanghui Jin, Xiaoqin Lu

**Affiliations:** 1 Department of General Practice, School of General Practice and Continuing Education, Capital Medical University, No. 10, Xitoutiao, You An Men Wai, Fengtai District, Beijing, 100069 China; 2 Department of General Practice, Beijing Tongren Hospital, Capital Medical University, Beijing, China

**Keywords:** Primary care, Family doctor service, Quality of care, Process quality, Type 2 diabetes mellitus, Beijing

## Abstract

**Background:**

Family doctor service (FDS) is a scheme oriented to improving the access and continuity of primary care in China. Type 2 diabetes mellitus (T2DM) management is a core component of FDS. However, evidence on the quality of T2DM care is lacking and the potential association between FDS attributes and T2DM care is largely unknown. This study attempted to assess the process quality of T2DM care in general practice and explore the association between patient perceived FDS attributes and process quality of T2DM care.

**Methods:**

Total 400 patients were recruited from 5 community health service centers in two urban districts in Beijing. Questionnaire survey and extraction of data from electronic health record (EHR) were conducted to collect patient characteristics, patient perceived FDS attributes (accessibility, continuity and team-based care) and process quality indicators (monitoring and health counseling indicators). Chi-square test and a two-level generalized linear mixed model (GLMM) were used to explore the association between FDS attributes and process quality.

**Results:**

The utilization rate of all the 12 indicators in monitoring, 6 indicators in health counseling and all the 18 process indicators, was 12.8%, 23.8% and 6.0% respectively. Over half of the patients (56.8%) perceived all the 3 FDS attributes. There were statistically significant associations between accessibility of care and lipid (*p* = 0.008), electrocardiogram (*p* = 0.016), retinopathy (*p* = 0.037) and peripheral neuropathy (*p* = 0.006) monitoring and each of the 6 health counseling indicators (all the *p* values < 0.05). Regular follow up (*p* = 0.039), plasma blood glucose (*p* = 0.020), blood pressure (*p* = 0.026), body mass index (*p* = 0.044) and foot (*p* = 0.005) monitoring as well as each of the 6 health counseling indicators (all the *p* values < 0.05) were more likely to be received by patients when continuity of care was ensured. Patients who were managed by a GP team had higher utilization rate of glycosylated hemoglobin monitoring (*p* = 0.026) and each of the 6 health counseling indicators (all the *p* values < 0.05). When the patients perceived one more FDS attribute, the indicators they received significantly increased by 1.50 (coefficient = 1.50, *p* < 0.001). Patients between the age of 65 and 74 years received 1.15 more indicators than those under 65 (coefficient = 1.15, *p* = 0.003). Patients with more than ten years duration of T2DM received 0.74 more indicators (coefficient = 0.74, *p* = 0.028). Patients taking both insulin and oral medicine received 0.97 more indicators than those taking oral medication only (coefficient = 0.97, *p* = 0.027). Patients who were managed by GPs with on-job training experience received 1.19 more indicators (coefficient = 1.19, *p* = 0.040). Among the patients who had completed junior high school or below, having better self-report health status (≥ 60) received 2.40 less indicators (coefficient = -2.40, *p* = 0.004).

**Conclusions:**

Improvement of key monitoring and health counseling indicators might be needed in T2DM care in general practice in Beijing, China. Policies for improving process quality of T2DM care should be considered.

**Supplementary Information:**

The online version contains supplementary material available at 10.1186/s12875-022-01838-0.

## Background

A strong primary care delivery system is the foundation of an efficient health care system [1]. In China, primary care facilities refer to community health service centers (CHSCs) and community health service stations (CHSSs) in urban areas, as well as township hospitals and village clinics in rural areas. The Family Doctor Service (FDS) model was piloted in 2009 in the New Medical Reform and was officially implemented nationwide since 2016 in China [2, 3]. In the FDS model, patients can make a contract with the family doctor team, also known as general practitioner (GP) team, which usually composes of GP, nurse and preventive care physician [3]. The team provides basic medical and public health services as well as health management for contracted community residents [3]. FDS is a top-down policy in China, contract rate is a key performance indicator of FDS. The proportion of patients who sign a contract is related to the performance and payment of GP team [4]. The number of contracted patients is increasing significantly. The contract rate of the priority populations (e.g., the elderly, pregnant women, children, and people with chronic disease including diabetes) was 93.06% in Beijing in 2019 [5].

China has the highest number of people with diabetes (116.4 million, 8.3% of the national population) [6, 7], and type 2 diabetes mellitus (T2DM) management is a core component of FDS [3]. The content of FDS for T2DM patients includes personal health records, lifestyle assessment, follow-up, regular monitoring, health counseling, priority appointment and referral [8]. One important goal of FDS is to improve the quality of care in general practice, which is critical for preventing diabetes associated complications [9]. However, challenges still exist in providing high quality diabetes care in general practice. Most patients with diabetes have signed a contract with GP teams, while available large-scale epidemiological evidence pointed to substantial gaps in diabetes care in China [10]. A national survey enrolled 75,880 participants from 31 provinces of mainland China in 2017 found that only 40.9% of those with diabetes were being treated. And among patients treated, 49.4% had glycosylated hemoglobin (HbA1c) controlled [10]. Another national study reported that during 2011–2015, the recurrent hospital admission rate among people with diabetes steeply increased from 18.87% to 28.45% [11]. Health information technology systems for diabetes care are essential for continuity and coordination [12]. However, the electronic health record (EHR) in general practice is still fragmented and isolated in its ability to analyze and integrate comprehensive information about patient care in China [12]. Besides, development and deployment of health information technology system in general practice are highly decentralized, as a result, the systems in general practice are largely not interoperable with hospitals to facilitate patient referrals [13]. Although guidelines on the management of T2DM are available, GPs demonstrate low levels of clinical guideline knowledge and practice [14].

The Royal College of General Practitioners (RCGP) defined the quality in general practice as all GPs strive to provide the best possible care for patients, families and communities [15]. Quality assessment, including process quality assessment, can be useful to identify the deficiencies in quality of care and conducive to quality improvement [16]. Previous studies of T2DM quality measurement in primary care have been carried out by using process indicators (e.g., diabetes education and counselling, monitoring of HbA1c and cholesterol and screening for microvascular complications) in multiple countries [16–19]. These studies showed that there is variation in the process quality of diabetes care, and the process quality could be affected by patient characteristics (e.g., gender, comorbidities and frequency of consultation), GP characteristics (gender, lack of training and workload) and institution characteristics (e.g., poor availability of monitoring equipment and inadequate number of clinicians and nurses) [16–19]. Most of these studies focused on a biomedical understanding of quality (i.e., the conceptualization of a gold standard of quality guided by clinical guidelines). But patient’s perceptions of the care are likely to be key drivers of health service utilization as well [20]. For a comprehensive and detailed assessment of the quality of T2DM care, both clinical quality and patient’s perceptions of the health service need to be evaluated and then compared [20].

Despite large scale expansion of the FDS scheme and the fast increase of contract rate in China [21], clinical indicators of T2DM care are not systematically assessed in the pay for performance system of general practice. Evidence on the quality of T2DM care in general practice is lacking, and the potential association between patient perceived attributes of FDS and clinical quality of T2DM care is largely unknown. Given the poor data availability of intermediate quality indicators such as HbA1c and lipid results in the current EHR system in general practice in China, this study intended to assess T2DM care in terms of process quality. Perceived FDS attributes among contracted patients and the potential association between FDS attributes and the process quality of T2DM care were also explored.

## Methods

In the previous study, we have developed a set of quality indicators of T2DM care in general practice by an iterative Delphi process, which consisted of the following steps: (1) construct a preliminary list of indicators from the following sources: T2DM clinical guidelines and indicators produced by key organizations in China, USA, UK and Australia as well as published research papers on T2DM quality measurement in primary care (see Supplementary Table 1, Additional file 1); (2) refine the preliminary indicators by an expert consultation meeting; (3) identify quality indicators by two rounds of email-based Delphi survey and a face to face consensus meeting [22]. The final set of indicators were grouped into seven domains access, monitoring, health counseling, records, health status (e.g., HbA1c target, blood lipid target and hypoglycemia episodes), patient satisfaction and self-management [22]. Monitoring and health counseling indicators were used in this study to evaluate the process quality of T2DM care.

### Study design and participants

**Fig. 1:**
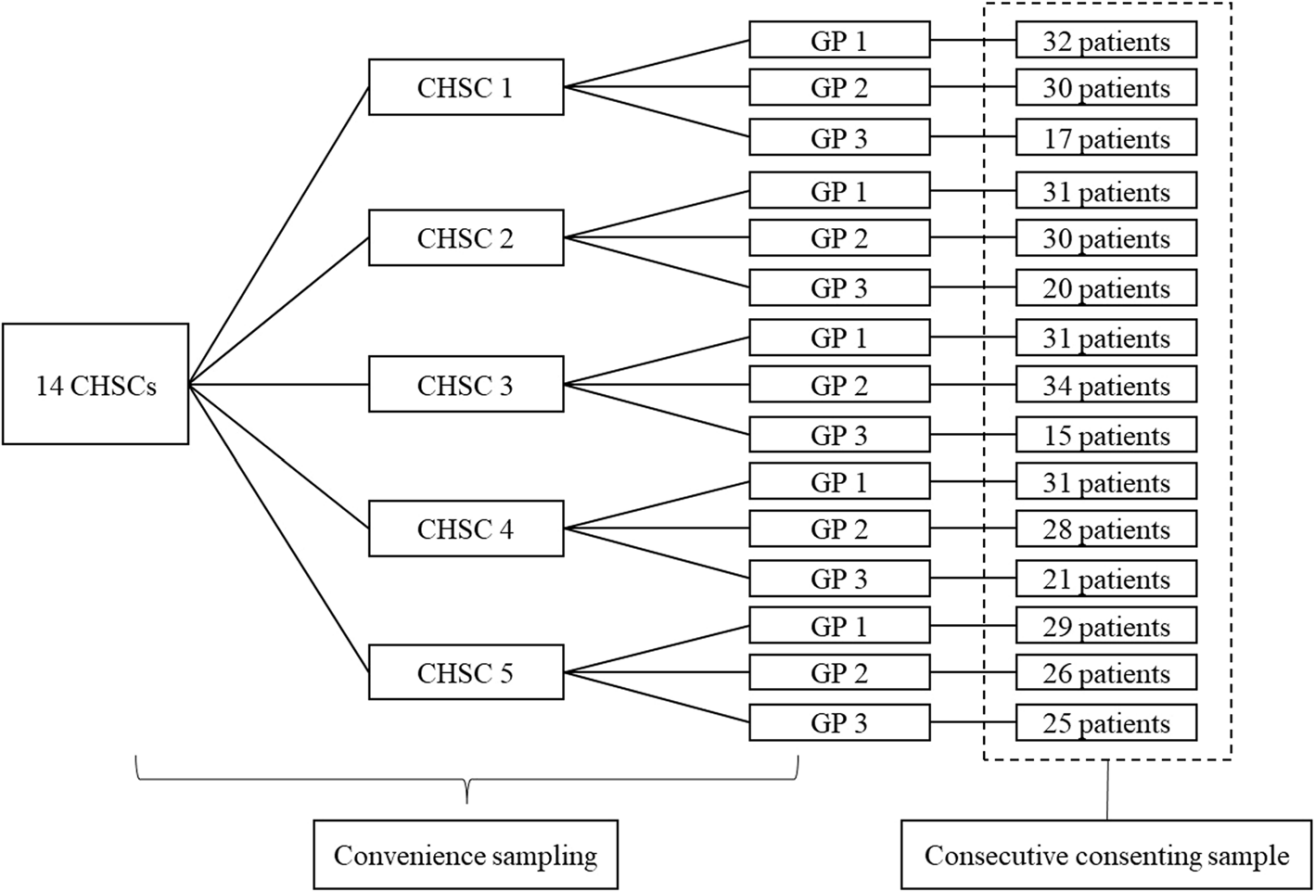
Flow chart of sample selection. From fourteen CHSCs that were teaching bases of Capital Medical University five CHSCs were chosen. There were 118 GPs in total in the selected five CHSCs and we had chosen three GPs per CHSC. The fifteen GPs selected have overall 5131 contracted T2DM patients. GP, general practitioner; CHSC, community health service center; T2DM, type 2 diabetes mellitus

We carried out a cross-sectional study from May to December 2019 in community health service centers in Beijing China. Non-probability sampling method was adopted as this was a preliminary study in the exploration of process quality of T2DM in primary care [23]. To ensure a stable and adequate amount of contracted T2DM patients, general practice experts and researchers were consulted before sampling. The sampling plan had a three-stage design. First, from fourteen CHSCs that were teaching sites of Capital Medical University, five CHSCs were chosen by convenience sampling considering the following criteria: (1) stable amounts (> 1000) of contracted T2DM patients; (2) had five or more GPs; (3) available for the study. Second, there were 118 GPs in total in the five CHSCs and we had chosen three GPs per CHSC by convenience sampling considering the following criteria: (1) worked in the community for over 5 years; (2) ranked above intermediate grade professional title; (3) had > 50 contracted T2DM patients; (4) available for the study. Third, consecutive patients visiting the selected GPs were invited and informed about the study. All those who were eligible and consented were recruited in the study as “consecutive consenting sample.” [24]. The inclusion criteria of patients were as follows: (1) diagnosed with T2DM for at least 1 year before the study; (2) signed a contract with the selected GPs; (3) being able to answer the questions clearly and logically. Total 400 patients (80 patients per CHSCs) were recruited in this study. These patients were from fifteen GPs in five CHSCs located in two of the six urban districts of Beijing. Please see Fig. 1. The sample size was chosen based on the available funding granted.

### Data collection

A pilot study had been conducted at two CHSCs in Beijing in April 2019 to test the feasibility of monitoring and health counselling indicators. We found that the information of partial indicators was not required by the FDS model (e.g., records of foot and retinopathy monitoring), which was not available or missing in the general practice EHR system. Indicators in the two domains were further modified (i.e., modification of data collection method and indicator content) based on general practice expert consultation and research team meeting to guarantee data credibility and availability (see Supplementary Table 2, Additional file 1). Revised process indicators were shown in Table 1. Combination of patient questionnaire and review of EHR was recommended to collect data of the monitoring and health counseling indicators. The questionnaire and EHR extraction form were revised through multiple rounds of research team meetings.

**Table 1:** Quality indicators used to assess performance

Process quality indicators	Measurement	Data source
Monitoring		
Regular follow up	The percentage of patients who are followed up for at least 4 times by the GP team in the preceding 12 months	EHR
Plasma blood glucose monitoring	The percentage of patients who have at least 4 measurements of plasma blood glucose test (fasting or post-prandial) by the GP team in the preceding 12 months	EHR
BP monitoring	The percentage of patients who have at least 4 measurements of BP by the GP team in the preceding 12 months	EHR
BMI monitoring	The percentage of patients who have at least 1 measurement of BMI in the preceding 12 months	EHR
Waist circumference monitoring	The percentage of patients who have at least 1 measurement of waist circumference in the preceding 12 months	EHR
HbA1c monitoring	The percentage of patients who have at least one measurement of HbA1c test in the preceding 12 months	Patient questionnaire
Foot monitoring	The percentage of patients who have at least one diabetic foot examination in the preceding 12 months Tests: Skin inspection, pulse palpation	Patient questionnaire
Lipid monitoring	The percentage of patients who have at least one measurement of lipid test in the preceding 12 months Tests: TC, TG, LDL-C, HDL-C	Patient questionnaire
Nephropathy monitoring	The percentage of patients who have at least one nephropathy examination in the preceding 12 months Tests: creatinine, blood urea nitrogen, urine protein	Patient questionnaire
ECG monitoring	The percentage of patients who have at least one measurement of ECG in the preceding 12 months	Patient questionnaire
Peripheral neuropathy monitoring	The percentage of patients who have at least one peripheral neuropathy examination in the preceding 12 months Tests: temperature, vibration sensation and pinprick sensation, 10-g monofilament testing	Patient questionnaire
Retinopathy monitoring	The percentage of patients who have at least1 retinopathy examination in the preceding 12 months Tests: vision, fundus cope or fundus photography	Patient questionnaire
Health counseling		
Diet counseling	The percentage of patients who are provided with diet counseling in the preceding 12 months	Patient questionnaire
Exercise counseling	The percentage of patients who are provided with exercise counseling in the preceding 12 months	Patient questionnaire
Psychological counseling	The percentage of T2DM patients who are provided with psychological assessment or referral to professional counseling when necessary in the preceding 12 months	Patient questionnaire
Hypoglycemia awareness counseling	The percentage of patients who are provided with hypoglycemia awareness counseling in the preceding 12 months	Patient questionnaire
Medication safety counseling	The percentage of patients who are provided with medication safety counseling in the preceding 12 months	Patient questionnaire
Emergency help counseling ^a^	The percentage of patients who are provided with emergency help counseling in the preceding 12 months	Patient questionnaire

*EHR* Electronic health record, *BP* Blood pressure, *BMI* Body mass index, *HbA1c* Glycosylated hemoglobin, *TC* Total cholesterol, *TG* Triglyceride, *LDL-C* Low density lipoprotein cholesterol, *HDL-C* High density lipoprotein cholesterol, *ECG* Electrocardiogram

^a^Emergency help counseling is provided for the patient to improve the patient’s knowledge on seeking emergency help

The questionnaire consisted of three sections. The first section contained questions regarding patient socio-demographic variables (i.e., gender, age, marital status and education level) and health statues (i.e., coexisting health problems, number of years since diagnosis, T2DM associated therapy, T2DM related visits and self-reported health status). Number of years since diagnosis was collected by patients’ self-reported information. Patients reported their overall health on the Visual Analogue Scale (EQ-VAS) based on their health on the day of survey, with anchor points 0 (‘worst imaginable health state’) and 100 (‘best imaginable health state’). In the second section, we used three adopted items to learn about patient perceived attributes of FDS (i.e., accessibility, continuity and team-based care) based on literature review and FDS related policies in China [3, 25]. The three items included: (1) is it accessible to get health care from general practice when needed? (2) is there a personal GP providing continuous care for you? (3) could you perceive a functioning GP team (including the GP, nurse, and preventive care physician)? The items were with ternary results ‘Yes’, ‘No’ or ‘Not sure’, and only if the answer is ‘Yes’ will the attribute be considered as perceived by patient. The third section investigated the process quality of T2DM care using 7 monitoring and 6 health counseling indicators (see Table 1). In order to reduce recall bias, patients just answered whether relevant quality indicators had been performed, not the frequency. The items of process quality indicators in patient questionnaire were with ternary results ‘Yes’, ‘No’ or ‘Not sure’ and only if the answer is ‘Yes’ will the service be considered as received by patients. In order to ensure good response rates to all questions and the exchange of information to reduce misunderstanding, we conducted the questionnaire survey through one-on-one, face-to-face interviews by five investigators (i.e., two PhD. candidate, two master candidates and one lecturer of general practice) in the waiting room before or after consultation. The investigators were mainly responsible for explaining the items to the patients and recording patients’ answers. Before the investigation, the investigators had received unified training by JGH (lecturer of general practice) on data collection, and potential problems that may be encountered during the investigation were also discussed.

EHR information extraction form consisted of patient’s basic information (i.e., name, age, gender) and the frequency of five process quality indicators (see Table 1). After completing the questionnaire survey, patients’ basic information was recorded into the EHR information extraction form. EHR database was reviewed to extract data by two investigators together (one master candidates and one lecturer of general practice) using the EHR information extraction form.

Information on the GPs, including age, gender, highest degree, working years, professional title, GP training experience, total number of contracted T2DM patients were collected. Written informed consent was obtained from each participant in this study.

#### Data analysis

Descriptive statistics were used to describe patient and GP characteristics, patient perceived attributes of FDS and process quality indicators. Performance of process quality indicators was compared among different FDS attributes by using Chi-square test.

Since we hypothesized that the patients within the same GP might get the chance of a similar quality of T2DM care compared to those from other GPs, the analysis involving only one level of data violates the assumptions of equal variance and independence of observations [26]. Thus, we used a two-level generalized linear mixed model (GLMM) to test the effect sizes of patient and GP-level factors on process quality of T2DM care. The patients comprise the first level, and the GPs comprise the second level. The model used maximum likelihood as estimation method and specified the random effects for GPs. Dependent variable was the performance of process quality, which was considered as continuous variables and calculated by adding up the indicators received by patient. We opted for running three models using identity link. Model-1 examined the effects of patient perceptions of FDS. To control potential confounding from patient socioeconomic and health status, we adjusted for patient characteristics in model 2. In model 3, we additionally controlled for GP characteristics. Variables both on patient and GP-level were considered for included as fixed effects in the models provided there was no evidence of collinearity. Collinearity was checked using the variance inflation factor (VIF). A VIF more than 5 is considered as cause for concern, and more than 10 almost certainly indicates a serious collinearity problem [27]. In this study the VIFs of all variables were less than 4 (ranging from 1.092 to 3.897) which means no collinearity. The variables at a *p*-value of < 0.05 was assessed. Patient education level and self-reported health status had a significant interaction, which was included in the GLMM.

The fixed effects estimated the relationship between quality performance and patient and GP-level factors expressed as coefficient with 95% confidence intervals (CIs). The random-effects estimate the variation in quality performance across different GPs expressed as Intra-Class Correlation (ICC) [26]. The models were diagnosed using the Bayesian's Information Criterion (BIC) and the Akaike’s Information Criterion (AIC). The model with smaller BIC and AIC fits the data better and confirms the goodness-of-fit [26].

Data were analyzed using SPSS (Statistical Package for the Social Science) for Windows, version 26.0 and all the tests were two sided. *P* < 0.05 was considered statistically significant. All methods were performed in accordance with the relevant guidelines and regulations.

## Results

### Characteristics of patients and GPs

Of the 400 patients, 58.0% were male, 79.8% were with a spouse and the average age was 68.9 ± 9.16 (ranging from 38 to 92) years. More than half of the patients (54.5%) completed high school or above. There were 90.2% of patients had at least one and 35.2% had three or more coexisting health problems. The median number of years since diagnosis (with 1 missing) was 10 (ranging from 1 to 59) years. The most common therapy was oral medication only (74.2%). The percent of patients who had more than 12 visits at the CHSC in the 12 months preceding investigation was 61.5% (with 3 missing) (see Table 2). All the 15 GPs were female with an average age of 42.5 ± 4.14 (ranging from 37 to 49) years. 10 GPs had a bachelor’s degree. The average working years was 16.3 ± 6.48 (ranging from 8 to 30) years. For each GP, the total number of contracted T2DM patients ranged from 120 to 539. The information of the 15 GPs was shown in Table 2.

**Table 2:** Patient socio-demographic and health statues characteristics and GP information

Characteristics	N	Percent (%)
Patient Characteristics (*N* = 400)		
Gender		
Male	168	42.0
Female	232	58.0
Age group (ranging from 38 to 92)		
< 65 years	137	34.2
65–74 years	155	38.8
≥ 75 years	108	27.0
Marital status		
with spouse	319	79.8
without a spouse	81	20.2
Education level		
Junior high school and below	182	45.5
High school and above	218	54.5
Coexisting health problems (ranging from 0 to 7)		
T2DM only	39	9.8
1–2	220	55.0
≥ 3	141	35.2
Number of years since diagnosis (ranging from 1 to 59)		
≤ 10 years	168	42.0
> 10 years	231	57.8
T2DM associated therapy		
Oral anti-diabetic medication only	297	74.2
Combination of oral medication and insulin	71	17.8
Others ^a^	32	8.0
Number of T2DM related visits per year ^b^		
≤ 12 times	151	37.8
> 12 times	246	61.5
Self-reported health status (ranging from 10 to 100)		
< 60	39	9.8
≥ 60	361	90.2
GP Characteristics (*N* = 15)		
Age group (ranging from 37 to 49)		
≤ 40 years	7	46.7
41 ~ 45 years	4	26.7
> 45 years	4	26.7
Female	15	100.0
Highest degree		
Master	4	26.7
Bachelor and below ^c^	11	66.7
GP working years (ranging from 8 to 30)		
≤ 10 years	4	26.7
11–15 years	5	33.3
> 15 years	6	40.0
Professional title		
Intermediate grade title	6	40.0
Senior grade title	9	60.0
GP training		
No training experience	4	26.7
On-job training	11	73.3
Number of contracted T2DM patients (ranging from 120 to 539)		
< 300	5	33.3
300–400	6	40.0
> 400	4	26.7

*GP* General practitioner, *T2DM* Type 2 diabetes mellitus

^a^Including physiotherapy, health products, lifestyle intervention and insulin only

^b^Ranges of T2DM related visit per year were collected, no specific numbers

^c^Ten GPs had a bachelor’s degree and one GP had a college degree

### Process quality and perceived FDS attributes

The median number of T2DM care received by patients was 9 (ranging from 0 to 12) in the dimension of monitoring, 4 (ranging from 0 to 6) in the dimension of health counseling, and 12 (ranging from 2 to 18) in total. The utilization rate of all the 12 indicators in monitoring, 6 indicators in health counseling and all the 18 process indicators, were 12.8%, 23.8% and 6.0% respectively. The least frequently received care was peripheral neuropathy examination (28.7%) in the monitoring domain and emergency help counseling (42.5%) in the health counseling domain. It was accessible for most patients (87.8%) to get health care from general practice. Most of the patients (86.5%) had a personal GP providing continuous care for them. There were 67.3% of the patients recognized that they were being managed by a GP team (see Table 3). The distribution of all patients was 3.5%, 8.3%, 31.5% and 56.8% for those perceived 0, 1, 2 and 3 attributes of FDS, respectively.

**Table 3:** Distribution of process quality indicators and FDS attributes (*N* = 400)

Items	Data source	N	Percent (%)
Process quality indicators			
Monitoring			
Regular follow up	EHR	304	76.0
Plasma blood glucose monitoring	EHR	283	70.8
BP monitoring	EHR	307	76.8
BMI monitoring	EHR	303	75.8
Waist circumference monitoring	EHR	378	94.5
Lipid monitoring	Patient questionnaire	374	93.5
ECG monitoring	Patient questionnaire	358	89.5
Nephropathy monitoring	Patient questionnaire	354	88.5
HbA1c monitoring	Patient questionnaire	335	83.8
Retinopathy monitoring	Patient questionnaire	250	62.5
Foot monitoring	Patient questionnaire	150	37.5
Peripheral neuropathy monitoring	Patient questionnaire	115	28.7
Health counseling			
Diet counseling	Patient questionnaire	306	76.5
Exercise counseling	Patient questionnaire	295	73.8
Hypoglycemia awareness counseling	Patient questionnaire	231	57.8
Psychological assessment or counseling	Patient questionnaire	204	51.0
Medication safety counseling	Patient questionnaire	193	48.3
Emergency help counseling	Patient questionnaire	170	42.5
FDS attributes			
Ensured access to general practice	Patient questionnaire	351	87.8
Continuous Care from a personal GP	Patient questionnaire	346	86.5
Care was provided in a team-based approach	Patient questionnaire	269	67.3

*FDS* Family doctor service, *EHR* Electronic health record, *BP* Blood pressure, *BMI* Body mass index, *ECG* Electrocardiogram, *HbA1c* Glycosylated hemoglobin, *GP* General practitioner

### Association between perceived FDS attributes and quality indicators performance

There were statistically significant associations between accessibility of care and lipid (*p* = 0.008), electrocardiogram (*p* = 0.016), retinopathy (*p* = 0.037) and peripheral neuropathy (*p* = 0.006) monitoring and each of the 6 health counseling indicators (all the *p* values < 0.05). Regular follow up (*p* = 0.039), plasma blood glucose (*p* = 0.020), blood pressure (*p* = 0.026), body mass index (*p* = 0.044) and foot (*p* = 0.005) monitoring as well as each of the 6 health counseling indicators (all the *p* values < 0.05) were more likely to be received by patients when continuity of care was ensured. Patients who were managed by a GP team had higher utilization rate of glycosylated hemoglobin monitoring (*p* = 0.026) and each of the 6 health counseling indicators (all the *p* values < 0.05) (see Table 4).

**Table 4:** Comparison of indicators performance between different patients’ perception of FDS attributes

	Ensured access to general practice, %			Continuous Care from a personal GP, %			Care was provided in a team-based approach, %		
Indicators	Yes (*N* = 351)	No/Not sure (*N* = 49)	*p *^*a*^	Yes (*N* = 346)	No/Not sure (*N* = 54)	*p *^*a*^	Yes (*N* = 269)	No/Not sure (*N* = 131)	*p *^*a*^
Monitoring									
Regular follow up	76.1	75.5	0.932	77.7	64.8	0.039^*^	77.3	73.3	0.374
Plasma blood glucose	71.5	65.3	0.371	72.8	57.4	0.020^*^	71.4	69.5	0.694
BP	76.4	79.6	0.615	78.6	64.8	0.026^*^	77.7	74.8	0.521
BMI	75.5	77.6	0.754	77.5	64.8	0.044^*^	77.0	73.3	0.422
Waist circumference	94.0	98.0	0.257^b^	95.1	90.7	0.193^b^	95.5	92.4	0.191
Lipid	94.9	83.7	0.008^b*^	94.2	88.9	0.238^b^	94.1	92.4	0.521
ECG	90.9	79.6	0.016^*^	90.2	85.2	0.266	90.3	87.8	0.435
Nephropathy	89.5	81.6	0.108	89.0	85.2	0.412	88.5	88.5	0.983
HbA1c	84.6	77.6	0.209	85.0	75.9	0.094	86.6	77.9	0.026^*^
Retinopathy	64.4	49.0	0.037^*^	63.6	55.6	0.257	64.3	58.8	0.283
Foot	39.0	26.5	0.090	40.2	20.4	0.005^*^	39.8	32.8	0.178
Peripheral neuropathy	31.1	12.2	0.006^*^	30.3	18.5	0.074	30.0	25.2	0.272
Counseling									
Diet	79.5	55.1	< 0.001^*^	80.6	50.0	< 0.001^*^	79.9	69.5	0.021^*^
Exercise	76.9	51.0	< 0.001^*^	78.0	46.3	< 0.001^*^	78.8	63.4	0.001^*^
Hypoglycemia	60.1	40.8	0.010^*^	61.0	37.0	0.001^*^	62.8	47.3	0.003^*^
Psychological	55.3	20.4	< 0.001^*^	56.4	16.7	< 0.001^*^	62.1	28.2	< 0.001^*^
Medication safety	51.3	26.5	0.001^*^	52.3	22.2	< 0.001^*^	54.3	35.9	0.001^*^
Emergency help	45.3	22.4	0.002^*^	46.8	14.8	< 0.001^*^	48.7	29.8	< 0.001^*^

*FDS* Family doctor service, *BP* Blood pressure, *BMI* Body mass index, *ECG* Electrocardiogram, *HbA1c* Glycosylated hemoglobin, *GP* General practitioner

^a^*p* value based on Chi-square test

^b^1 cells (25.0%) have expected count less than 5, and the value is taken from continuity correction

^*^p < 0.05

### Influencing factors on process quality performance of T2DM care

The AIC and BIC values for the three models were 2067.69/2079.59, 2003.16/2014.93, and 1988.78/2000.48, respectively. The results indicated that the full model (Model-3) with the smallest AIC and BIC values fitted the data better than the other models. The ICC in Model-3 indicated that 2.4% (ICC: 0.024) of the variation in the quality performance was attributable to differences across GPs.

The fixed effects of Model-3 showed that one more FDS attribute perceived by patients, indicators received by patients significantly increased by 1.50 (coefficient = 1.50, 95%CI 1.08,1.92, *p* < 0.001) after patient and GP characteristics were adjusted. Several non-modifiable patient and GP factors also had statistically significant influence on the process quality performance. Patients between the age of 65 and 74 years received 1.15 more indicators than those under 65 (coefficient = 1.15, 95%CI 0.40,1.90, *p* = 0.003). Patients with more than ten years duration of T2DM reported receiving 0.74 more indicators (coefficient = 0.74, 95%CI 0.08,1.41, *p* = 0.028). Patients who took both insulin and oral medicine received 0.97 more indicators, comparing with those who took oral medication only (coefficient = 0.97, 95%CI 0.11,1.83, *p* = 0.027). Patients who were managed by GPs with on-job training experience received 1.19 more indicators (coefficient = 1.19, 95%CI 0.06,2.32, *p* = 0.040). The *p*-value for the interaction of education level and self-reported health status was 0.023 (see Table 5). Among the patients who had completed junior high school or below, having better self-report health status (≥ 60) received 2.40 less indicators (coefficient = -2.40, 95%CI -4.01,-0.78, *p* = 0.004). However, among the patients who had completed high school and above, there was not significant association between self-reported health status and quality performance (see Table 6).

**Table 5:** Generalized linear mixed models of predictors of process quality of T2DM care

Variables	Model 1		Model 2		Model 3	
	coefficient (95% CI)	*p*	coefficient (95% CI)	*p*	coefficient (95% CI)	*p*
Patient-level variables						
Gender						
Male	-	-	0 (reference)		0 (reference)	
Female			0.54 (-0.09,1.18)	0.093	0.52 (-0.12,1.16)	0.113
Age group						
< 65 years			0 (reference)		0 (reference)	
65–74 years			1.16 (0.41,1.91)	0.002^*^	1.15 (0.40,1.90)	0.003^*^
≥ 75 years			0.69 (-0.17,1.55)	0.114	0.61 (-0.26,1.47)	0.169
Marital status						
With spouse	-	-	0 (reference)		0 (reference)	
Without a spouse			-0.47 (-1.27,0.32)	0.242	-0.50 (-1.29,0.30)	0.223
Coexisting health problems						
0–2			0 (reference)		0 (reference)	
≥ 3			0.22 (-0.45,0.90)	0.521	0.19 (-0.49,0.87)	0.580
Number of years since diagnosis						
≤ 10 years	-	-	0 (reference)		0 (reference)	
> 10 years			0.79 (0.13,1.45)	0.019^*^	0.74 (0.08,1.41)	0.028^*^
T2DM associated therapy						
Oral anti-diabetic medication only	-	-	0 (reference)		0 (reference)	
Combination of oral medication and insulin			0.88 (0.02,1.73)	0.044^*^	0.97 (0.11,1.83)	0.027^*^
Others ^a^			-0.69 (-1.88,0.50)	0.256	-0.68 (-1.88,0.51)	0.263
Number of T2DM related visits per year						
≤ 12 times	-	-	0 (reference)		0 (reference)	
> 12 times			0.46 (-0.20,1.12)	0.171	0.50 (-0.18,1.17)	0.147
Education level * Self-reported health status	-	-	-	0.025^*^	-	0.023^*^
Number of perceived FDS attributes	1.57 (1.17,1.97)	< 0.001^*^	1.49 (1.08,1.90)	< 0.001^*^	1.50 (1.08,1.92)	< 0.001^*^
GP-level variables						
Age group						
36–40 years	-	-	-	-	0 (reference)	
41–45 years					0.58 (-0.91,2.07)	0.444
> 45 years					0.17 (-1.6,1.94)	0.852
Highest degree						
Master (ref)	-	-	-	-	0 (reference)	
Bachelor and below					0.11 (-0.83,1.04)	0.821
GP working years						
≤ 10 years	-	-	-	-	0 (reference)	
11–15 years					-0.50 (-2.72,1.72)	0.655
> 15 years					-0.22 (-3.12,2.68)	0.881
Professional title						
Intermediate grade title	-	-	-	-	0 (reference)	
Senior grade title					0.83 (-1.11,2.78)	0.401
GP training						
No training experience	-	-	-	-	0 (reference)	
On-job training					1.19 (0.06,2.32)	0.040^*^
Number of contracted T2DM patients						
< 300	-	-	-	-	0 (reference)	
300–400					-0.33(-1.36,0.70)	0.532
> 400					-0.66(-1.71,0.40)	0.223
GP-level variance (SE)	0.155 (0.211)		0.214 (0.229)		0.222 (0.438)	
ICC	0.015		0.023		0.024	
AIC	2067.69		2003.16		1988.78	
BIC	2079.59		2014.93		2000.48	

*FDS* Family doctor service, *GP* General practitioner, *ICC* Intra-Class Correlation, *BIC* Bayesian's Information Criterion, *AIC* Akaike’s Information Criterion

^a^Including physiotherapy, health products, lifestyle intervention and insulin only

^*^p < 0.05

**Table 6:** Interaction of patient education level and self-reported health status on the performance of process quality

Education level	Self-reported health status	coefficient (95% CI)	*p* ^a^
Junior high school and below	< 60	0 (reference)	
	≥ 60	-2.40 (-4.01, -0.78)	0.004^*^
High school and above	< 60	0 (reference)	
	≥ 60	-0.01 (-1.37, 1.34)	0.984

^a^Estimated effects were adjusted on the fully adjusted model 3 ^*^p < 0.05

## Discussion

This study attempted to assess the process quality of T2DM care and perceived FDS attributes among contracted patients in urban general practices in Beijing, and explore the potential association between patient perceived FDS attributes and the process quality of T2DM care. We found that there was variation in the performance of different process quality indicators. The main deficiencies were peripheral neuropathy examination (28.7%) in monitoring and emergency help counseling (42.5%) in health counseling. Among the 3 FDS attributes, team-based care was perceived by the fewest number of patients (67.3%). There was significant association between the performance of process quality of T2DM care and patient perceived FDS attributes. Characteristics of GP (training experience) and patient (age, time since diagnosis, T2DM associated therapy, and education level * self-reported health status) also significantly influenced the process quality performance.

In this study, routine lipid, nephropathy and HbA1c monitoring were received by over 80% patients, which was in line with previous studies by Sugiyama, et al. in Japan (HbA1c test 96.7%) [28], Canedo, et al. in America (cholesterol test 85.4%) [29], Tran et al. in Norway (HbA1c test 94.7%, cholesterol test 94.2%) [30] and Somanawat et al. in Thailand (microalbuminuria test 81.7%) [16]. However, retinopathy (62.5%), foot (37.5%), and peripheral neuropathy (28.7%) monitoring were performed poorly and the utilization rate was lower than in other countries (foot exam 69%, dilated eye exam 64.9% in America and retinopathy screening 91.1%, foot exam 95.7% in Thailand) [16, 29]. The variation in process quality could be derived from multiple causes. First, in China, it is convenient for patients to have blood tests and the expenses can be reimbursed by medical insurance [31]. However, primary care resources in community are relatively insufficient for the screening of T2DM complications including retinopathy, foot, and peripheral neuropathy monitoring [32]. Second, due to the lack of community medical resources, patients need to be referred to higher-level medical institutions for examination. However, the referral mechanism has not been effectively established in China [33], and the health information technology systems in general practice are largely not interoperable with hospitals to support the referrals [13]. Third, payment for performance as a main part of a GP’s income (about 30%) were mostly determined by the quantity (e.g., the indicator of FDS contract rate set by the government) rather than the clinical quality of care [4, 34]. Increasing FDS contract rate of T2DM patients has become an important goal in general practice. However, there is a lack of systematic performance measurement and reward mechanism regarding the monitoring of diabetes related complications [13]. This may be one of the causes for the suboptimal performance in key indicators (e.g., retinopathy, foot, peripheral neuropathy monitoring). Health counseling indicators in our study, such as diet counseling (79.5%) and exercise counseling (76.9%) were performed better than another national survey in China (diet 65.0%, exercise 49.0%) [11], the main reason might be that the general practice in CHSC in Beijing were better developed and can provide relatively high-quality services, compared with other regions in China [35]. There was still room for improvement, especially in medication safety (48.3%) and emergency help counseling (42.5%). In China, the workload of GPs is relatively heavy, and there is little time for health counseling during consultation [36]. Previous study also showed that communication between GPs and T2DM patients was inadequate [37]. The suboptimal education and training of providers may be one of the main reasons as well [13].

The recommended care processes examined in this study are recognized as being central to the management of T2DM, especially the prevention of complications. Our results suggested that patient’s perception of FDS attributes (accessibility, continuity and team-based) contributed to the recommended processes of care. This is similar with the study by Arah et al., which indicated better patient experience was associated with more self-reporting recommended care [38]. Successful management of T2DM partly bases on securing patient compliance and trust [38]. Although accessibility, continuity and team-based are not service attributes that are clinically necessary, they may all influence patients’ trust and their willingness to return for or adhere to treatment, which in turn might improve health-care outcomes substantially [38]. Accessibility of medical facilities and health care providers provides structural support for complication monitoring and patient consultation. GPs providing continuous care for a given patient have a greater understanding of the patient’s health issues than multiple providers do and can provide better care [39]. Besides, continuous care helps to increase the familiarity of GPs and patients, which may in turn encourages GPs to provide more health counseling to the patient [40]. The addition of nurse and preventive care physician might be able to make up for the large workload of GPs. However, in this study, more than 30% of patients did not perceive a functioning GP team. There are still barriers to build effective GP team, such as increased workload, insufficient staffing as well as internal team cooperation problems [41].

The results of GLMM also showed several non-modifiable patient factors were associated with the process of T2DM care, which is in line with previous researches [17, 18, 42]. Patients with older age or longer time since diagnosis were more likely to receive more indicators of diabetes care in this study, which is in line with study by Van, et al. [17]. Generally, T2DM takes years to cause diabetes related organ damage and it is unlikely to find foot, eye or kidney damage in patients with the onset of diagnosis. Risk of complications increases with age or the duration of T2DM [43]. Therefore, patients with older age or longer history of T2DM may be more likely to do tests to monitor complications. This study found that the patient education level was a significant interaction for the effect of self-reported health status on quality performance. When patients with lower education felt that they were in better health status, they tend to ignore the health service utilization. Previous studies also showed patients with higher education level tended to have higher health literacy which had a positive correlation with health service utilization [44, 45].

Although CHSCs have already made strides in important areas of the patient-centered aspects of FDS in our study, further research is needed to evaluate ongoing efforts to sophisticate the model of FDS. Policy implications can be considered upon the results of this study. First, sheer effort on increasing FDS contract rate i.e., the quantity of care will not be sufficient, more focus should be put on the quality of care including key FDS attributes [21]. The shortage of diverse multidisciplinary GP teams persisted, which limited a functional response to the needs of community [13]. Even with a massive expansion of the primary care medical workforce, GPs alone cannot address all the support needs of the huge T2DM population in China. Evidence based multi-professional team model at primary care level should be encouraged [37]. Second, patients with older age or longer time since diagnosis were more likely to received more T2DM care given the risk of complications increases with age or the duration of the disease [17]. However, one may not be aware how long T2DM has been existing exactly. Even if the patient has a long history of the disease, they could be less likely to benefit from T2DM care and there might be other more important health issues than diabetes near the end of their life span. Health care providers should understand patient within his/her unique context, respond to patient needs and values, and provide him/her with individualized care [46]. Third, patients' health literacy can influence their service utilization [44]. Health education should be strengthened to improve patients' health literacy, especially for patients with lower education level, thereby ensuring the effective delivery of diabetes care. Lastly, health information technology can help GPs to monitor patients efficiently and accurately, and patients can also depend on the system to manage their disease [47]. Many countries use comprehensive medical information system to evaluate the quality of diabetes care to increase the feasibility and efficiency of evaluation [42]. However, there are still significant gaps in the development of medical information system of CHCS in China, impeding the data extraction of quality indicator. Further improvement is needed to promote data sharing and comprehensive analysis. The main purpose of quality evaluation is to improve the quality of care. Quality improvement initiatives in the medical field are widely carried out [48]. However, there are few studies on the quality improvement in general practice in China yet [49]. GPs should lead the GP team and cooperate with patients and stakeholders to establish a quality improvement culture in general practice, and use evidence-based quality improvement methods [48].

### Strengths and limitations

Our study represents one of the first attempts to assess the process quality of T2DM care by using detailed indicators based on clinical guidelines and explore the association between clinical quality and patient perception of care in China. The results of our study indicated the significant association between patient perception of FDS attributes and process quality of T2DM care, which might support the importance of patient perceived experience in delivering and measuring the quality of T2DM care. However, there are some limitations. Firstly, the study adopted non-probability sampling with a relatively small sample size, which could affect the precision of statistical analysis. All the fifteen GPs included in this study were female because most GPs who met the inclusion criteria were female in those CHSCs selected. This may be one reason for that only 2.4% (ICC: 0.024) of the variation in the quality performance was attributable to differences across GPs. Due to the explorative nature of this preliminary study, the findings based on non-probability sampling can only be generalized to the population from which the sample is drawn and not to the entire population. Future studies with random sampling frames can enhance the external validity of the findings. Nevertheless, we made attempt to control for the characteristics of participants to provide the clearest possible image of quality of T2DM care in CHSCs in Beijing, China. Secondly, quality indicators used in this study were constructed with only monitoring and health counseling indicators due to the limited availability of more quality-related information in the EHR system. Thus, these indicators may not be an exact representation of all the quality-related aspects. Monitoring and health counseling as indicators for the T2DM care in our study should be interpreted with prudence, since repeating counseling annually may be unnecessary for patients with well-controlled diabetes. And patients near the end of their live span may be less likely to benefit from these services. Thirdly, the information collected through questionnaire survey was self-reported in the past year and patients’ incomprehension of the quality indicators as well as recall biases might induce underreported information of process quality. In order to reduce these biases, the investigator fully explained the meaning of each indicator to the participants and they just answered whether they had received the relevant services, not the frequency. There may be a social desirability bias relying on self-report data, as participants may be inclined to present themselves in a more positive light or appease the course leader. In order to ensure the authenticity of the patient's answer, before the survey, we explained to patients the importance of accurate answers and promised them that data was fully anonymized for the purposes of research access and all identifiable data would be removed. The study variables were reports asking patients whether it happened, not evaluations asking them to rate what happened. What’s more, we set up a "Not sure" option, which is convenient for patients to give a negative answer in a euphemistic way. On the other hand, we conducted the questionnaire survey through interviews by five investigators. Interviewer bias and recording errors could be a possible limitation. The questionnaire was revised through multiple rounds of research team meetings. We also provided careful training for investigators about the principles of questionnaire survey and information recording to ensure the consistency of data collection. Finally, even though we had adapted a two-level GLMM to test the effect sizes of patient and GP-level factors on process quality of T2DM care, there were factors may have been neglected, such as patient’s diabetes knowledge and compliance and CHSCs’ human resources. Future research is needed to identify more variables to explain the quality of T2DM care in general practice in China. As with any observational study causality cannot be determined.

## Conclusions

Variation was found in the performance of regular monitoring and health counseling indicators. Improvement of key indicators might be needed in T2DM care in urban general practices, Beijing, China. The main deficiencies were peripheral neuropathy examination in monitoring and emergency help counseling in health counseling. Patient perceived FDS attributes (i.e., accessibility, continuity and team-based care) were significantly associated with process quality of T2DM care in this study.

## Supplementary Information

**Additional file 1: Supplementary Table 1.** Sources of guidelines and indicators from key institutions reviewed. **Supplementary Table 2.** Modification of the indicators in domains of monitoring and health counseling.

## Data Availability

The data used and/or analyzed during the current study are available from the corresponding author on reasonable request.

## Abbreviations

CHSC: Community health service center
CHSS: Community health service station
FDS: Family doctor service
GP: General practitioner
T2DM: Type 2 diabetes mellitus
BP: Blood pressure
BMI: Body mass index
HbA1c: Glycosylated hemoglobin
TC: Total cholesterol
TG: Triglyceride
LDL-C: Low density lipoprotein cholesterol
HDL-C: High density lipoprotein cholesterol
ECG: Electrocardiogram
EHR: Electronic health record
GLMM: Generalized linear mixed model
CI: Confidence intervals
