# Supplementary material for: Process quality of type 2 diabetes mellitus care and association with patient perceived attributes of family doctor service in urban general practices, Beijing, China
Source: BMC Prim Care. 2022 Sep 7;23:228. doi: 10.1186/s12875-022-01838-0 (PMC9454121; doi:10.1186/s12875-022-01838-0)
Supplement: Supplementary file 1 — Additional file 1: Supplementary Table 1. Sources of guidelines and indicators from key institutions reviewed. Supplementary Table 2. Modification of the indicators in domains of monitoring and health counseling. [file 12875_2022_1838_MOESM1_ESM.docx]

**Additional File**

Supplementary Table 1, Additional file 1. DOC. Sources of guidelines and indicators from key institutions reviewed.

Supplementary Table 2, Additional file 1. DOC. Modification of the indicators in domains of monitoring and health counseling.

**Supplementary Table 1.** Sources of guidelines and indicators from key institutions reviewed

| Type of sources | Sources | Institution | Publication year | Country |
| --- | --- | --- | --- | --- |
| Clinical guidelines | Guidelines on the prevention and treatment of T2DM in China 2017[1] | Chinese Medical Association, Diabetes Chapter | 2017 | China |
|  | National guidelines on prevention and treatment of diabetes in primary care [2] | Chinese Medical Association, Diabetes Chapter | 2018 | China |
|  | Standards of medical care in diabetes-2018 [3] | American Diabetes Association | 2018 | USA |
|  | Type 2 diabetes in adults: management [4] | National Institute for Health and Care Excellence | 2015 | UK |
|  | Global guideline for type 2 diabetes [5] | International Diabetes Federation | 2005 | Not applicable |
|  | General practice management of type 2 diabetes 2016–18 [6] | The Royal Australian College of General Practitioners | 2016 | Australia |
| Indicators from key institutions | Community health service quality standard [7] | Community Health Association of China | 2016 | China |
|  | Quality and outcomes framework [8] | National Health Service | 2018 | UK |
|  | Primary care measures [9] | The Centers for Medicare & Medicaid Services | 2016 | USA |
|  | Clinical indicators for Australian general practice [10] | The Royal Australian College of General Practitioners | 2015 | Australia |

Abbreviation: T2DM Type 2 diabetes mellitus; USA United States of America; UK United Kingdom

1. Chinese Diabetes Mellitus Association. Guidelines on the prevention and treatment of type 2 diabetes mellitus in China 2017 [in Chinese]. Chin J Diabetes Mellitus. 2018;10(1):4–66

2. Chinese Diabetes Mellitus Association. National guidelines for the prevention and control of diabetes in primary care (2018) [in Chinese]. Chin J Intern Med. 2018;57(12):885–93.

3. American Diabetes Association. Standards of medical care in diabetes-2018. Diabetes Care. 2018;41(Suppl 1):S1–153.

4. National Institute for Health and Care Excellence. Type 2 diabetes in adults: management. 2015. https://www.nice.org.uk/guidance/ng28. Accessed 17 Mar 2022.

5. International Diabetes Federation. Global guideline for type 2 diabetes.43. 2012. https://www.idf.org/e-library/guidelines/79-global-guideline-for-type-2-diabetes.html. Accessed 17 Mar 2022.

6. Royal Australian College of General Practitioners. General practice management of type 2 diabetes 2016–18. 2016. <https://www.racgp.org.au/FSDEDEV/media/documents/Clinical%20Resources/Guidelines/Diabetes/General-practice-management-of-type-2-diabetes_1.pdf>. Accessed 17 Mar 2022.

7. Community Health Association of China. Community health service quality standards [in Chinese]. 2016. <https://www.chs.org.cn/news/show/60/>. Accessed 17 Mar 2022.

8. National Health Service. Quality and outcomes framework 2018/19. 2019.

https://digital.nhs.uk/data-and-information/publications/statistical/quality-and-outcomes-framework-achievement-prevalence-and-exceptions-data/2018-19-pas. Accessed 17 Mar 2022.

9. Centers for Medicare & Medicaid Services. Primary care measures. 2016.https://www.cms.gov/Medicare/Quality-Initiatives-Patient-Assessment-Instruments/QualityMeasures/Core-Measures.html. Accessed 17 Mar 2022.

10. The Royal Australian College of General Practitioners. Clinical indicators for Australian general practice. 2015. [https://www.racgp.org.au/running-a-practice/practice-management/general-practice-governance/clinical-indicators. Accessed 17 Mar 2022](https://www.racgp.org.au/running-a-practice/practice-management/general-practice-governance/clinical-indicators.%20Accessed%2017%20Mar%202022).

**Supplementary Table 2.** Modification of the indicators in domains of monitoring and health counseling

| Process quality indicators | Measurement | Data source | Modification |
| --- | --- | --- | --- |
| Monitoring |  |  |  |
| Regular follow up | The percentage of patients who are followed up for at least 4 times by the GP team in the preceding 12 months | EHR | - |
| Plasma blood glucose monitoring | The percentage of patients who have at least 4 measurements of plasma blood glucose test (fasting or post-prandial) by the GP team in the preceding 12 months | EHR | - |
| BP monitoring | The percentage of patients who have at least 4 measurements of BP by the GP team in the preceding 12 months | EHR | - |
| BMI monitoring | The percentage of patients who have at least 1 measurement of BMI in the preceding 12 months | EHR | - |
| Waist circumference monitoring | The percentage of patients who have at least 1 measurement of waist circumference in the preceding 12 months | EHR | - |
| HbA1c monitoring ^a b^ | The percentage of patients who have at least two measurements of HbA1c test in the preceding 12 months | EHR | 1.Modify the frequency of test  2. Modify data source |
| Foot monitoring ^b^ | The percentage of patients who have at least one diabetic foot examination in the preceding 12 months  Tests: Skin inspection, pulse palpation | EHR | Modify data source |
| Lipid monitoring ^b^ | The percentage of patients who have at least one measurement of lipid test in the preceding 12 months  Tests: TC, TG, LDL-C, HDL-C | EHR | Modify data source |
| Nephropathy monitoring ^b^ | The percentage of patients who have at least one nephropathy examination in the preceding 12 months  Tests: creatinine, blood urea nitrogen, urine protein | EHR | Modify data source |
| ECG monitoring ^b^ | The percentage of patients who have at least one measurement of ECG in the preceding 12 months | EHR | Modify data source |
| Peripheral neuropathy monitoring ^b^ | The percentage of patients who have at least one peripheral neuropathy examination in the preceding 12 months  Tests: temperature, vibration sensation and pinprick sensation, 10-g monofilament testing | EHR | Modify data source |
| Retinopathy monitoring ^b^ | The percentage of patients who have at least1 retinopathy examination in the preceding 12 months  Tests: vision, fundus cope or fundus photography | EHR | Modify data source |
| Health counseling |  |  |  |
| Smoking counseling ^c^ | The percentage of T2DM patients who are provided with smoking assessment or referral to professional counseling when necessary in the audit year | Patient questionnaire | Delate |
| Diet counseling | The percentage of patients who are provided with diet counseling in the preceding 12 months | Patient questionnaire | - |
| Exercise counseling | The percentage of patients who are provided with exercise counseling in the preceding 12 months | Patient questionnaire | - |
| Psychological counseling | The percentage of T2DM patients who are provided with psychological assessment or referral to professional counseling when necessary in the preceding 12 months | Patient questionnaire | - |
| Hypoglycemia awareness counseling | The percentage of patients who are provided with hypoglycemia awareness counseling in the preceding 12 months | Patient questionnaire | - |
| Medication safety counseling | The percentage of patients who are provided with medication safety counseling in the preceding 12 months | Patient questionnaire | - |
| Emergency help counseling ^d^ | The percentage of patients who are provided with emergency help counseling in the preceding 12 months | Patient questionnaire | - |

Abbreviations: EHR Electronic health record; BP Blood pressure; BMI Body mass index; HbA1c Glycosylated hemoglobin; TC Total cholesterol; TG Triglyceride; LDL-C Low density lipoprotein cholesterol; HDL-C High density lipoprotein cholesterol; ECG Electrocardiogram

Note: ^a^ In order to reduce recall bias, patients just answered whether relevant quality indicators had been performed, not the frequency.

^b^ Detailed information of these seven indicators was not available or missing in the EHR system. Data were collected by patient questionnaire.

^c^ Considering smoking counseling is not an indicator measurable for all T2DM patients, and only 66 (16.5%) patients were identified as smoking in this study. The indicator of smoking counseling was not included in the analysis.

^d^ Emergency help counseling is provided for the patient to improve the patient’s knowledge on seeking emergency help.
